# Molecular mechanisms of neuroprotective effect of rutin

**DOI:** 10.3389/fphar.2025.1599167

**Published:** 2025-06-03

**Authors:** Zhang Chunmei, Wang Shuai

**Affiliations:** ^1^ School of Basic Medical, Luoyang Polytechnic, Luoyang, China; ^2^ Henan Provincial Engineering Research Center for Key Biomaterials in Immunology Technology, Luoyang, China; ^3^ Shenzhen Traditional Chinese Medicine Hospital, Shenzhen, China

**Keywords:** rutin, antioxidant, anti-inflammatory, neuroprotective effects, mechanisms, medicinal potential

## Abstract

Neurological diseases, including stroke, Alzheimer’s disease, Parkinson’s disease, and diabetic neuropathy, pose a significant global health burden. The rising incidence of these diseases, driven by factors including an aging population, lifestyle changes, and environmental influences, has intensified the urgent need for effective neuroprotective therapies. Rutin, a natural flavonoid glycoside widely distributed in various plants including buckwheat, citrus fruits, and onions, has garnered significant attention as a promising neuroprotective agent. This review comprehensively evaluates the current research on rutin’s multifaceted neuroprotective mechanisms, which encompass antioxidant, anti-inflammatory, anti-apoptotic, antidepressant, anticonvulsant, and analgesic effects, as well as its role in enhancing neural signal transduction, improving learning and memory, and protecting the blood-brain barrier. However, despite its broad spectrum of neuroprotective effects and favorable safety profile, the clinical application of rutin is currently limited by its relatively low bioavailability. To address this limitation and fully harness rutin’s therapeutic potential, future research should prioritize the development of innovative formulations designed to enhance its bioavailability.

## 1 Introduction

The nervous system includes the central nervous system (CNS) and the peripheral nervous system (PNS). It is reported that common neurological diseases worldwide include stroke, neonatal encephalopathy, migraine, Alzheimer’s disease (AD), Parkinson’s disease (PD) and diabetic neuropathy (Guo X et al., 2025). The common clinical manifestations of nervous system diseases include disturbance of consciousness, cognitive impairment, headache, vertigo, visual impairment, hearing impairment, motor impairment, somatosensory impairment and balance disorder. With the growth and aging of the global population, as well as the increase of risk factors such as environment, metabolism and lifestyle, the number of people suffering from stroke, AD, meningitis and other neurological diseases and deaths from these diseases has increased significantly (Mead et al., 2023; Calabrese et al., 2024; Mckee and Daneshvar, 2015).

**FIGURE 1:**
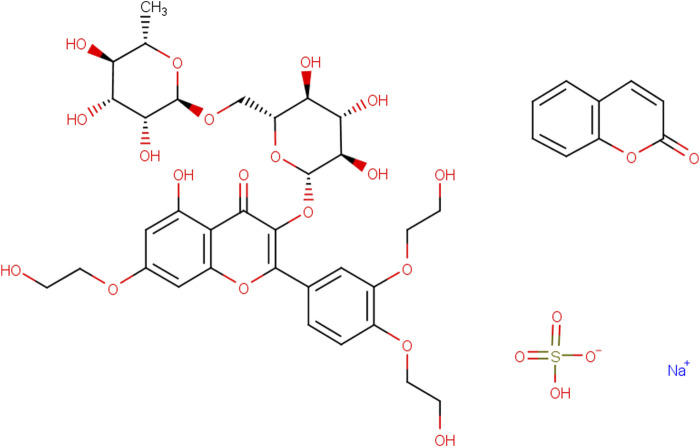
Structural formula of Rut.

Rutin (Rut), a natural flavonoid glycoside, is a light yellow or light green crystalline powder (Figure 1). It widely exists in Rut leaves, tobacco leaves, jujubes, apricots, orange peel, tomatoes, buckwheat flowers and other plants (Forouzanfar et al., 2025a; Nicola et al., 2024; Shimazu et al., 2021). The content of Rut in the flower buds of Sophora japonica is high up to 20% and Sophora japonica is often used as raw material for industrial extraction of Rut (Saafan et al., 2023). Rut has a variety of pharmacological effects such as antioxidant, anti-inflammatory, antihypertensive, maintaining vascular elasticity and neuroprotection (Mani et al., 2025; Abdullah et al., 2025; Jang et al., 2014). Due to its high pharmacological activity, less adverse drug reactions and wide range of targets, Rut is used to treat nervous system diseases (Chekuri et al., 2025; Forouzanfar et al., 2025b). Thus, Rut has a variety of neuroprotective mechanisms, including antioxidant effect, inhibition of neuronal apoptosis, improvement of learning and memory ability, protection of blood-brain barrier (BBB), which can be used as an effective drug for the treatment of nervous system diseases (Hai et al., 2024; Faysal et al., 2025; Xinghua et al., 2023; Xianchu et al., 2022). Rut also could pass through the BBB to improve the function of the hypothalamus pituitary adrenal axis by regulating the levels of monoamine neurotransmitters and brain-derived neurotrophic factors (BDNF) in the body, and exert neuroprotective effects on GABAergic, glutamatergic and cholinergic nervous systems (Motamedshariaty et al., 2014; Liu et al., 2021; Ji et al., 2024). As a safe and efficient new plant medicine, Rut has significant biological activity and has a wide application prospect in the medical field. This article will discuss the research progress of Rut neuroprotective effect based on the neuroprotective mechanisms.

## 2 Neuroprotective mechanisms of Rut

### 2.1 Antioxidant

Under physiological conditions, reactive oxygen species (ROS) produced by the body can be eliminated by antioxidant systems such as superoxide dismutase (SOD) and glutathione peroxidase (GSH PX) in the body. The process of generation and elimination is in dynamic balance to maintain the stability of the internal environment (Song et al., 2018; Nkpaa and Onyeso, 2018; Aldian et al., 2025). Under pathological conditions, the rate of ROS generation by the body is far higher than the rate of endogenous elimination, resulting in a large amount of ROS accumulation, so that DNA, protein, lipid and other macromolecular compounds in the cell are in a state of peroxidation and cannot play their normal physiological functions (Andonova et al., 2025; de Carvalho et al., 2025).

**FIGURE 2:**
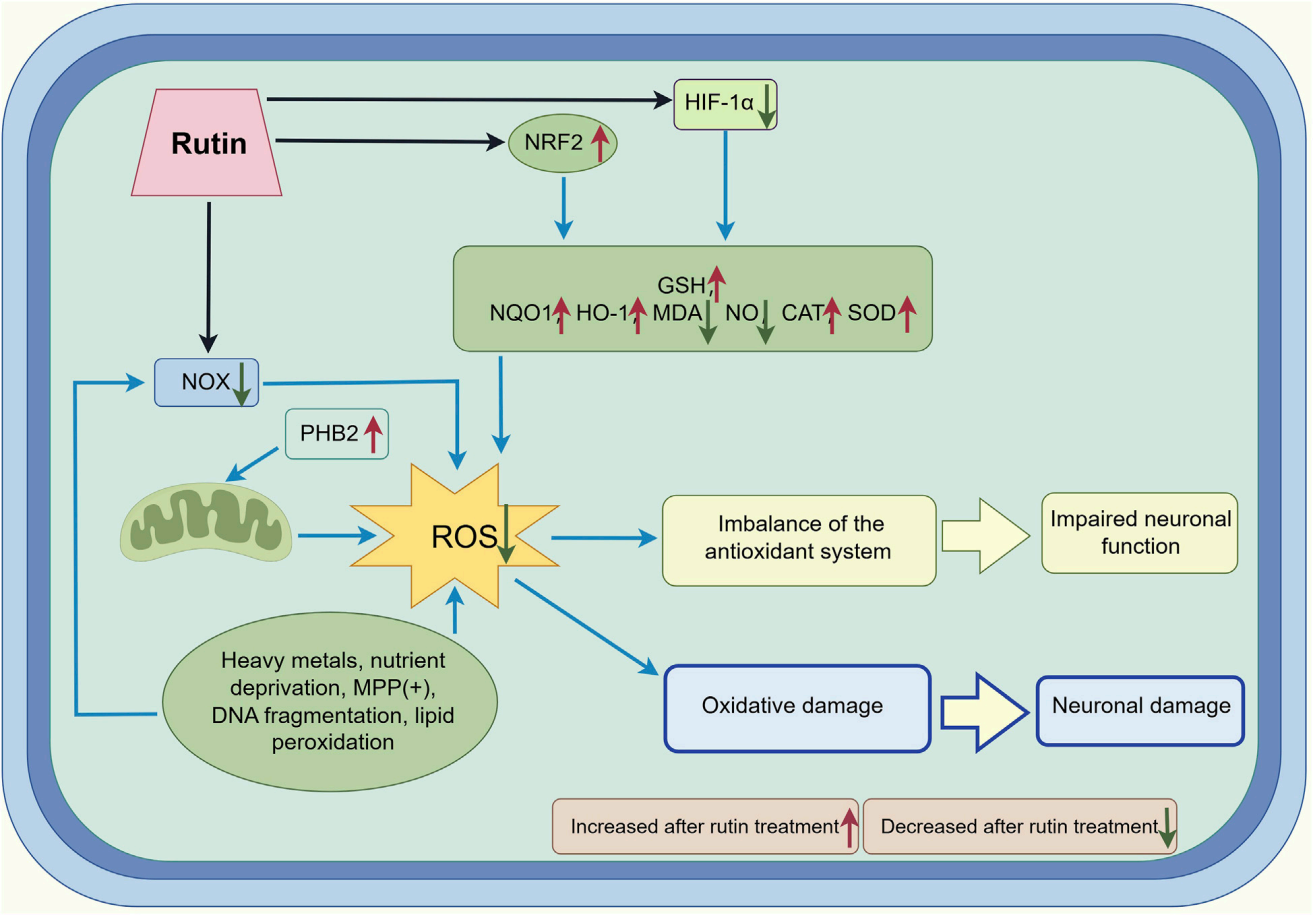
This figure illustrates the mechanisms of oxidative stress regulation and neuronal damage by Rut. In the presence of stressors such as heavy metals, nutrient deprivation, MPP(+), DNA fragmentation and lipid peroxidation, NOX and mitochondrial complex I PHB2 contribute to the generation of ROS. The imbalance of the antioxidant system, including decreased levels of GSH, NRF2, NQO1, HO-1 and increased levels of MDA, NO, CAT and SOD results in impaired neuronal function. Rut treatment modulates oxidative damage and neuronal injury by bidirectionally regulating key factors of the redox system, including upregulating antioxidant markers (GSH, NRF2, NQO1, HO-1) while downregulating oxidative stress indicators (MDA, NO). The figure highlights the role of Rut in modulating oxidative stress.

Nassiri-Asl M studied the effect of Rut on DNA oxidative damage in cultured rat pheochromocytoma cells (PC12) under nutrient deprivation. The results showed that Rut could improve the viability of PC12 cells under nutrient deprivation, reduce ROS production and lipid peroxidation (Nassiri-Asl et al. 2020). Lai X transfected Prohibitin 2 (PHB2) siRNA lentiviral particles into SH-SYSY cells treated with matrix metalloproteinase (MPP) (+) and found that Rut had a protective effect in PHB2 cells, but was inhibited in PHB2 silenced cells, suggesting that Rut may play its neuroprotective role by reducing oxidative damage and the depolarization level of mitochondrial membrane potential through PHB2 mediated mitochondrial autophagy (Lai et al. 2023). Arowoogun J established a rat model induced by copper. The levels of malondialdehyde (MDA) and nitric oxide (NO) in rat brain increased, and the activities of SOD and catalase (CAT) decreased, damaging neurons. Rut administration can improve nerve injury and regulate the levels of these biomarkers, suggesting that Rut can play a neuroprotective role by participating in antioxidant pathways and inhibiting oxidative stress (Arowoogun et al. 2021). Li F established the lead induced SH-SYSY cell model. After Rut intervention, it significantly reduced the oxidative stress of cells, promoted the translocation of nuclear factor red cell 2 related factor 2 (Nrf2) from the cytoplasm to the nucleus, and then activated the expression of antioxidant substances including Heme Oxygenase-1 (HO-1), and knocked out Nrf2 by small interfering RNA (siRNA) transfection to reduce the protective effect of Rut on lead induced cells, suggesting that Rut alleviated lead induced oxidative stress by activating Nrf2/ARE pathway in SH-SY5Y cells and played its antioxidant role (Li et al. 2024). Zamani K have studied that Rut can effectively relieve the symptoms of abnormal sciatic nerve pain in rats with chronic constrictive injury (CCI) and regenerate the sciatic nerve, suggesting that Rut can play its antioxidant role by increasing antioxidant mediators *in vivo* (Zamani et al. 2025). Uthra C studied the effect of Rut on oxidative damage of neurons induced by acrylamide, and found that Rut can reduce oxidative stress caused by acrylamide, suggesting that Rut plays its antioxidant role and has a significant protective effect on DNA oxidative damage caused by acrylamide (Uthra et al. 2022) (Figure 2; Table 1).

**TABLE 1:** Antioxidantion effects and its mechanism of Rut.

Experimental models	Description	Observed effects	Key targets/Pathways	References
Rats’ spinal cord injury model	the neuroprotective mechanisms of Rut for spinal cord injury	Neuroprotective effects through anti-oxidation, anti-inflammation and inhibition of p38 MAPK pathway. Inhibited p38 MAPK pathway	Anti-oxidation, anti-inflammation and inhibition of p38 MAPK pathway	Song et al. (2018)
Fluoride treated rats	neurobehavioral deficits, oxidative stress, neuro-inflammation and apoptosis in fluoride treated rats	neurobehavioral deficits, oxidative stress, neuro-inflammation and apoptosis	Neurobehavioral deficits, oxidative stress, neuro-inflammation, apoptosis	Nkpaa and Onyeso (2018)
PC12 cell under nutrient deprivation	the effect of Rut on DNA oxidative damage	Rut improved cell viability, reduced ROS production and lipid peroxidation	ROS, lipid peroxidation	Nassiri-Asl et al. (2020)
SH-SYSY cells treated with MPP (+) and transfected with PHB2 siRNA lentiviral particles	Rut had a protective effect in PHB2 cells but was inhibited in PHB2 silenced cells	Rut attenuates oxidative stress via PHB2-mediated mitophagy in MPP + -induced SH-SY5Y cells	PHB2 mediated mitochondrial autophagy	Lai et al. (2023)
Rat model induced by copper	Copper increased MDA and NO levels in rat brain, decreased SOD and CAT activities	Rut improved nerve injury, regulated biomarker levels	MDA/NO SOD/CAT	Arowoogun et al. (2021)
Lead induced SH-SYSY cell model	Rut intervention reduced oxidative stress, promoted Nrf2 translocation and activated HO-1 expression	Rut protects against lead-induced oxidative stress in SH-SY5Y cells	Nrf2/ARE pathway HO-1	Li et al. (2024)
Rats with (CCI)	Rut can relieve sciatic nerve pain symptoms and regenerate the sciatic nerve	Rut can play its antioxidant role by increasing antioxidant mediators *in vivo*	Antioxidant mediators	Zamani et al. (2025)
Neurons induced by acrylamide	Rut can reduce oxidative stress caused by acrylamide	Rut has a has a significant protective effect on DNA oxidative damage caused by acrylamide	DNA oxidative damage	Uthra et al. (2022)

### 2.2 Anti-inflammatory

Inflammatory response is a crucial factor in the pathogenesis of neurodegenerative diseases. Excessive neuroinflammation can exacerbate the damage to nerve cells, thereby further promoting the occurrence of neurological diseases (Taşlı et al. 2018). Neuroinflammatory response is one of the important triggers of neurodegenerative diseases. As the main immune cells in the CNS, microglia play a crucial role in maintaining brain homeostasis, including synaptic remodeling, BBB regulation, and neuroinflammation regulation (Hao et al. 2016; Sreelatha et al. 2024). When an inflammatory response occurs, microglia are immediately activated, and the activated microglia mainly have two states: M1 type and M2 type. M1 type microglia can cause local or widespread central nervous system damage by releasing cytotoxic substances and inflammatory factors, while M2 type microglia play a neuroprotective role by secreting anti-inflammatory mediators and neurotrophic factors (Sui et al. 2022; Jasim et al. 2025; Meimei et al. 2024). Therefore, targeting the selective activation of microglia, studying their functions and related inflammatory signaling pathways is of great significance for the treatment of nervous system inflammation and neurodegenerative diseases.

**FIGURE 3:**
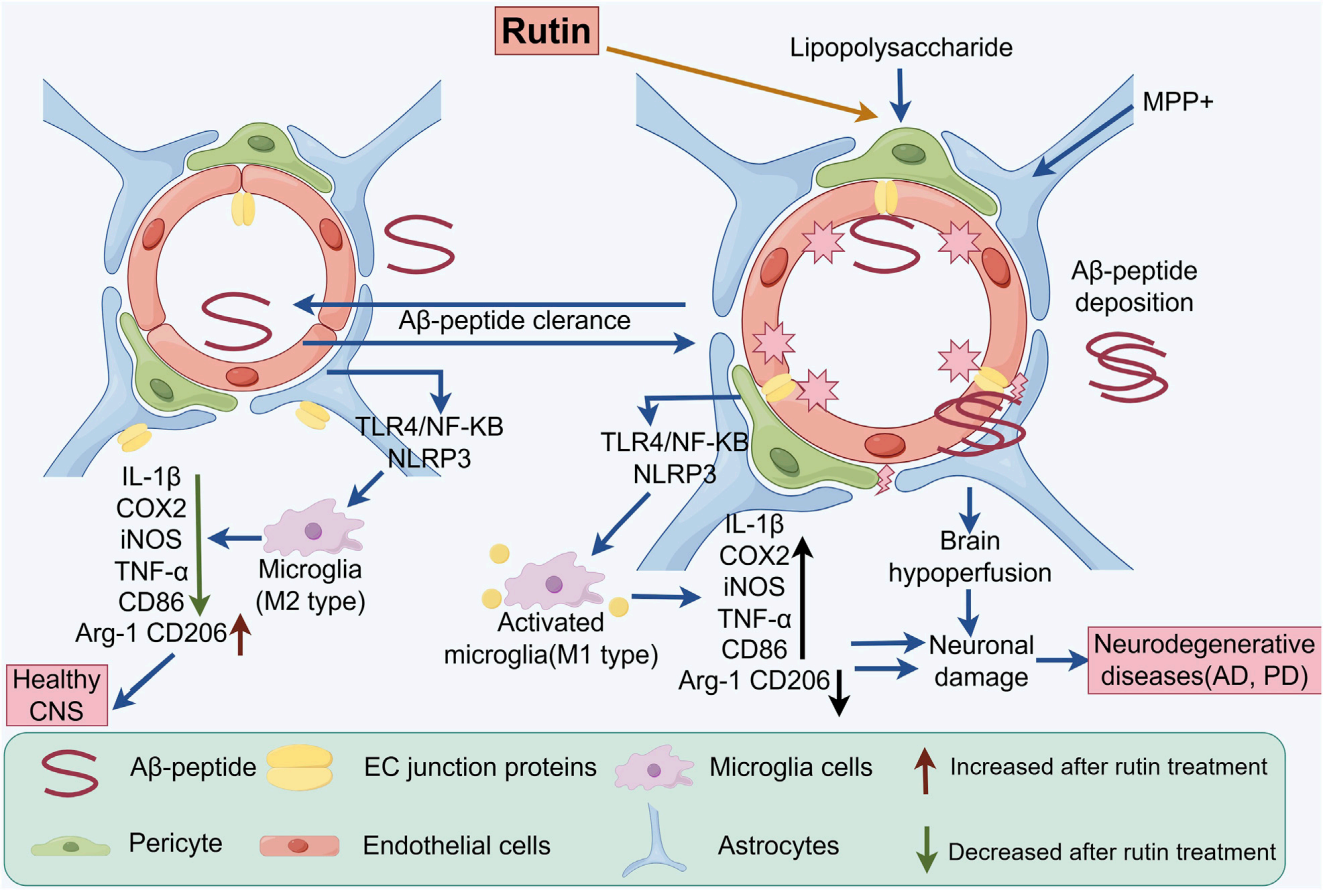
This figure reveals important factors in anti-inflammatory signaling pathway of Rut in the CNS microenvironment. Rut regulates the expression of pro-inflammatory factors such as IL-1β, COX2, iNOS and TNF-α by acting on signaling pathways such as TLR4/NF-κB and NLRP3 inflammasome. Rut can also promote the clearance of A β peptide, maintain the stability of β-catenin, and protect the tight junctions between cells from damage. In microglia, Rut may alleviate inflammatory response and promote tissue repair by regulating its polarization state (M1 to M2 transition).

Xu PX found that Rut exerts neuroprotective effects by reducing the levels of oligomeric Aβ, inhibiting the activation of glial cells, and decreasing the production of inflammatory cytokines, thereby alleviating memory impairment in AD transgenic mice (Xu et al., 2014). Khan MM observed that administration of Rut resulted in a decrease in the number of dopamine D2 receptors in the striatum due to an increase in glutathione and its dependent enzymes (glutathione peroxidase and glutathione reductase), dopamine, and its metabolite 3,4-dihydroxyphenylacetic acid, demonstrating the neuroprotective effect of Rut on PD (Khan et al. 2012). In addition, Javed H observed a reduction in “neuroinflammation” in a rat model of “Alzheimer’s type sporadic dementia”, and observed that Rut intervention alleviated impaired proliferation of hippocampal dentate gyrus (DG) cells and protected mice from morphological changes in the CA3 region, reversing cognitive deficits and improving memory ability in it (Javed et al. 2012). In cell experiments, Rut can inhibit the secretion of pro-inflammatory mediators such as nitric oxide (NO), tumor necrosis alpha, cyclooxygenase-2 (COX-2), nuclear factor kappa B (NF-κB), interleukin-1β (IL-1β), and alleviate lipopolysaccharide induced neuronal inflammation (Lee et al. 2024). In addition, it was found that Rut can also downregulate the expression of M1 type markers inducible nitric oxide synthase and CD86 in BV-2 microglia by regulating the Toll like receptor 4 (TLR4)/NF-κB signaling pathway, and increase the expression of M2 type markers arginase 1 (Arg1) and CD206, thereby promoting the transformation of BV-2 cells from M1 to M2 type and exerting anti-inflammatory effects at the cellular level (Hu et al. 2023) (Figure 3; Table 2).

**TABLE 2:** Anti-inflammatory effects of Rut.

Experimental models	Description	Observed effects	Key targets/Pathways	References
Methanol induced acute toxic optic neuropathy model	the protective effects of Rut	Rut reduced oxidative stress and provided neuroprotection	RAGE-NF-κB inflammatory signaling pathway	Taşlı et al. (2018)
Experimental rat model of subarachnoid hemorrhage	Rut’s effects on neuroinflammation	Rut inhibited neuroinflammation and provided neuroprotection	RAGE-NF-κB inflammatory signaling pathway	Hao et al. (2016)
Scopolamine-induced deficits model	neuroprotective properties of Rut Hydrate	Rut Hydrate reduced oxidative stress and provided neuroprotection	BDNF/TrkB/ERK/CREB/Bcl2 pathways	Sreelatha et al. (2024)
RAW 264.7 cells treated with LPS	Anti-inflammatory effects of Rut	Rut reduced NO production and COX-2 levels	NF-κB pathway	Lee et al. (2024)
Zebrafish models	Rut hydrate’s effects on neuroinflammation	Rut hydrate reduced neuroinflammation	NF-κB pathway	Hu et al. (2023)
Alzheimer’s disease transgenic mice model	Rut’s effects on spatial memory	Rut improved spatial memory by reducing Aβ oligomer level	Aβ oligomer level	Xu et al. (2014)
Animal model of Parkinson’s disease	Rut’s effects on dopaminergic neurons	Rut protected dopaminergic neurons from oxidative stress	Oxidative stress	Khan et al. (2012)
Rat model of sporadic dementia of Alzheimer type	Rut’s effects on cognitive impairments	Rut prevented cognitive impairments by ameliorating oxidative stress and neuroinflammation	Neuroinflammation	Khan et al. (2012)
Neuroinflammation models focusing on microglial activation	the regulation of microglial-mediated neuroinflammation by Flavonoids	Rut regulated microglial-mediated neuroinflammation	TLR4/NF-κB/NLRP3 pathway	Jasim et al. (2025)
Neuroinflammation models focusing on microglial activation	the effects of Rut on aging behaviors and neuroinflammation	Rut inhibited microglia activation	TLR4/NF-κB/NLRP3 pathway	Meimei et al. (2025)

### 2.3 Anti-apoptotic effect

**FIGURE 4:**
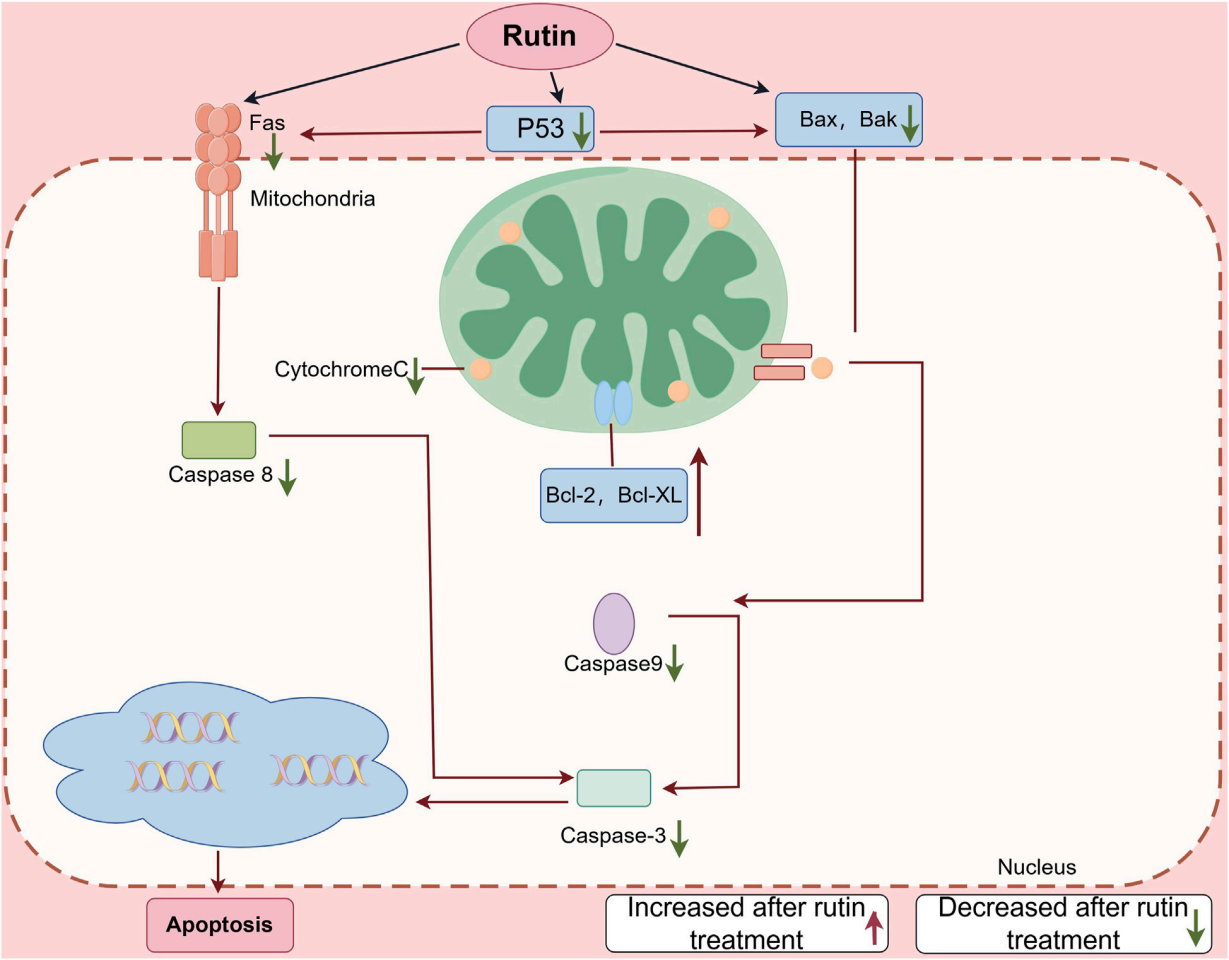
This figure shows the apoptotic signaling cascades triggered by Rut in nerve cells. Rut initiates the extrinsic apoptotic pathway through interaction with the Fas receptor, culminating in Caspase-8 activation. In parallel, Rut stimulates the intrinsic mitochondrial pathway by upregulating the tumor suppressor protein P53, which subsequently enhances the expression of the pro-apoptotic proteins Bax and Bak. The subsequent activation of Bax and Bak facilitates the release of cytochrome C from the mitochondria, thereby activating the Caspase cascade involving Caspase-9 and Caspase-3. Additionally, Rut downregulates the anti-apoptotic proteins Bcl-2 and Bcl-XL, thereby potentiating the apoptotic response. The convergence of these pathways leads to Caspase-3 activation, which is pivotal in executing apoptosis.

Apoptosis is an autonomous programmed cell death controlled by apoptotic genes. It is one of the types of programmed cell death, and its purpose is to maintain the stability of the human environment. The physiological process of apoptosis involves the activation, expression and regulation of a series of genes, such as the pro apoptotic gene B-cell lymphoma-2 associated X protein (Bax), cystainaspartate protease (Caspase), anti apoptotic gene B-cell lymphoma-2 (Bcl-2), tumor suppressor gene Tumor Protein 53 (p53) and myelocytomatosis oncogene (c-Myc) (Ma et al. 2025). Rut can reduce sodium nitroprusside induced injury to PC12 neurons by activating extracellular regulated protein kinase (ERK), reduce apoptosis triggered by nitric oxide synthase (NOS) producing NO, and protect neurons (Wang et al. 2015). Rut has a protective effect on SH-SYSY cells treated with 1-methyl-4-phenylpyridine ion (MPP (+)), which can inhibit cell apoptosis and reduce cell oxidative damage (Enogieru et al. 2021). Bilateral common carotid artery ligation and middle cerebral artery occlusion are common models for establishing cerebral ischemia in mice. Rut can not only improve the survival rate of mice after bilateral common carotid artery ligation, but also significantly reduce brain edema and the number of apoptotic neurons (Pu et al. 2007). Khan MM have shown that Rut can inhibit the apoptosis of ischemic nerve cells by reducing the expression of p53, preventing the morphological changes of nerve cells and improving the activity of endogenous antioxidant enzymes (Khan et al. 2009). Ischemic stroke, also known as cerebral infarction, refers to a cerebrovascular disease in which cerebral blood supply insufficiency is caused by stenosis or occlusion of cerebral blood supply arteries (carotid artery and vertebral artery), and ischemic necrosis of brain tissue is caused by cerebral ischemia and hypoxia, especially apoptosis of neurons after ischemia. Nkpaa KW studied Rut attenuates fluoride induced toxicity in the cerebrum and striatum of rats via mechanisms involving neuro-inflammation and anti-apoptosis in rats. Rut may be used as a neuroprotective agent against induced neurotoxicity through anti-apoptosis pathway (Nkpaa and Onyeso, 2018). In addition, Rut can significantly avoid the production of ROS and inhibit cell apoptosis through two different mechanism pathways (p38MAPK and JNK signaling pathway) (Li et al. 2017). Rut decreases the insulin amyloid fibrils-induced Neuro-2a cytotoxicity by reducing ROS levels which in turn downregulates Bax and upregulates Bcl-2 and pBad proteins (Mahendra et al. 2020). Rut can alleviate the neuronal injury in AD-like learning and memory impairment rat model by inhibiting reduce the neuronal apoptosis rate and the expression of apoptosis related proteins Bax in hippocampus (Sreelatha et al. 2024). Rut also significantly inhibit the activation of MAPK signaling pathway induced by cerebral ischemia-reperfusion, reduce the expression of apoptosis related proteins and the occurrence of apoptosis, indicating that Rut can play an anti apoptotic role in reducing nerve injury by inhibiting MAPK signaling pathway (Zhang et al. 2013) (Figure 4; Table 3).

**TABLE 3:** Anti-apoptotic effect of Rut.

Experimental models	Description	Observed effects	Key targets/Pathways	References
Chordoma cell lines	The study investigates the role of the lncRNA NEAT1/iASPP pathway in chordoma	Proliferation suppression and apoptosis induction in chordoma cells	NEAT1, iASPP, proliferation, apoptosis	Ma et al. (2025)
PC12 cells treated with sodium nitroprusside	Examines the protective effects of Rut on PC12 cells against sodium nitroprusside-induced neurotoxicity	Rut protects PC12 cells by activating PI3K/Akt/mTOR and ERK1/2 pathways	PI3K/Akt/mTOR, ERK1/2	Wang et al. (2015)
SH-SY5Y cells exposed to MPP+	Evaluates the effects of Rut on MPP + -exposed SH-SY5Y cells	Rut inhibits γH2AX and COX-2 and regulates antioxidant enzymes	γH2AX, COX-2, antioxidant enzymes	Enogieru et al. (2021)
Rats subjected to repeated cerebral ischemia	Investigates the neuroprotective effects of quercetin and Rut on spatial memory impairment and neuronal death in rats	Rut exhibits neuroprotective effects against spatial memory impairment and neuronal death	Spatial memory, neuronal death	Pu et al. (2007)
Rats with transient focal ischemia	Assesses the protective effects of Rut on neural damage induced by transient focal ischemia in rats	Rut protects against neural damage induced by transient focal ischemia	Neural damage, transient focal ischemia	Khan et al. (2009)
Rats treated with fluoride	Analyzes the effects of Rut on neurobehavioral deficits, oxidative stress, neuro-inflammation, and apoptosis in fluoride-treated rats	Rut attenuates neurobehavioral deficits, oxidative stress, neuro-inflammation, and apoptosis	Neurobehavioral deficits, oxidative stress, neuro-inflammation, apoptosis	Nkpaa and Onyeso (2018)
Neonatal rats exposed to isoflurane	Studies the effects of Rut on isoflurane-induced neuroapoptosis in the hippocampi of neonatal rats	Rut attenuates isoflurane-induced neuroapoptosis via JNK and p38 MAPK pathways	JNK, p38 MAPK	Li et al. (2017)
Neuro-2a cells exposed to insulin amyloid fibrils	Explores the mechanism of Rut-mediated inhibition of insulin amyloid formation and protection of Neuro-2a cells from fibril-induced apoptosis	Rut inhibits insulin amyloid formation and protects Neuro-2a cells from apoptosis	Insulin amyloid formation, apoptosis	Mahendra et al. (2020)
Scopolamine-induced deficits in BDNF/TrkB/ERK/CREB/Bcl2 pathways	Examines the neuroprotective properties of Rut hydrate against scopolamine-induced deficits	Rut hydrate exerts neuroprotective effects against scopolamine-induced deficits	BDNF/TrkB/ERK/CREB/Bcl2 pathways	Sreelatha et al. (2024)
Cerebral ischemia-reperfusion injury model	Evaluates the protective effect of flavonoid-rich extract from Rosa laevigata Michx on cerebral ischemia-reperfusion injury	The extract suppresses apoptosis and inflammation, providing protection against cerebral ischemia-reperfusion injury	Apoptosis, inflammation	Zhang et al. (2013)

### 2.4 Antidepressant effect

The hypothalamic pituitary adrenal (HPA) axis is associated with a variety of emotional and cognitive disorders. The HPA axis is hyperactive in patients with major depression, and the onset of the disease is closely related to the increase of cortisol content and the decrease of the activity of mineralocorticoid receptor and glucocorticoid receptor. Excessive content of endothelin in the body will stimulate hypothalamic atrophy and cause anxiety and emotional tension (Sălcudean et al. 2025). Schloms L found that Rut can effectively prevent the changes of adrenocorticotropic hormone in plasma and cortisol in serum of depression model mice, significantly reduce the cortisol level in adrenal H295R cells, and show antidepressant effect in open field test, forced swimming test and sucrose preference test (Schloms et al. 2014). Dexamethasone is a synthetic glucocorticoid, which can cause the death of hippocampal CA3 neurons, and then cause learning and memory disorders. The water maze test showed that the escape latency of Rut pretreatment group was significantly lower than that of dexamethasone group, and Rut pretreatment could effectively change the morphological damage of CA3 area caused by dexamethasone. This study suggests that Rut may regulate the activity of HPA axis by affecting the glucocorticoid level *in vivo*, and then participate in neuroprotection and play an antidepressant role (Tongjaroenbuangam et al. 2011).

**FIGURE 5:**
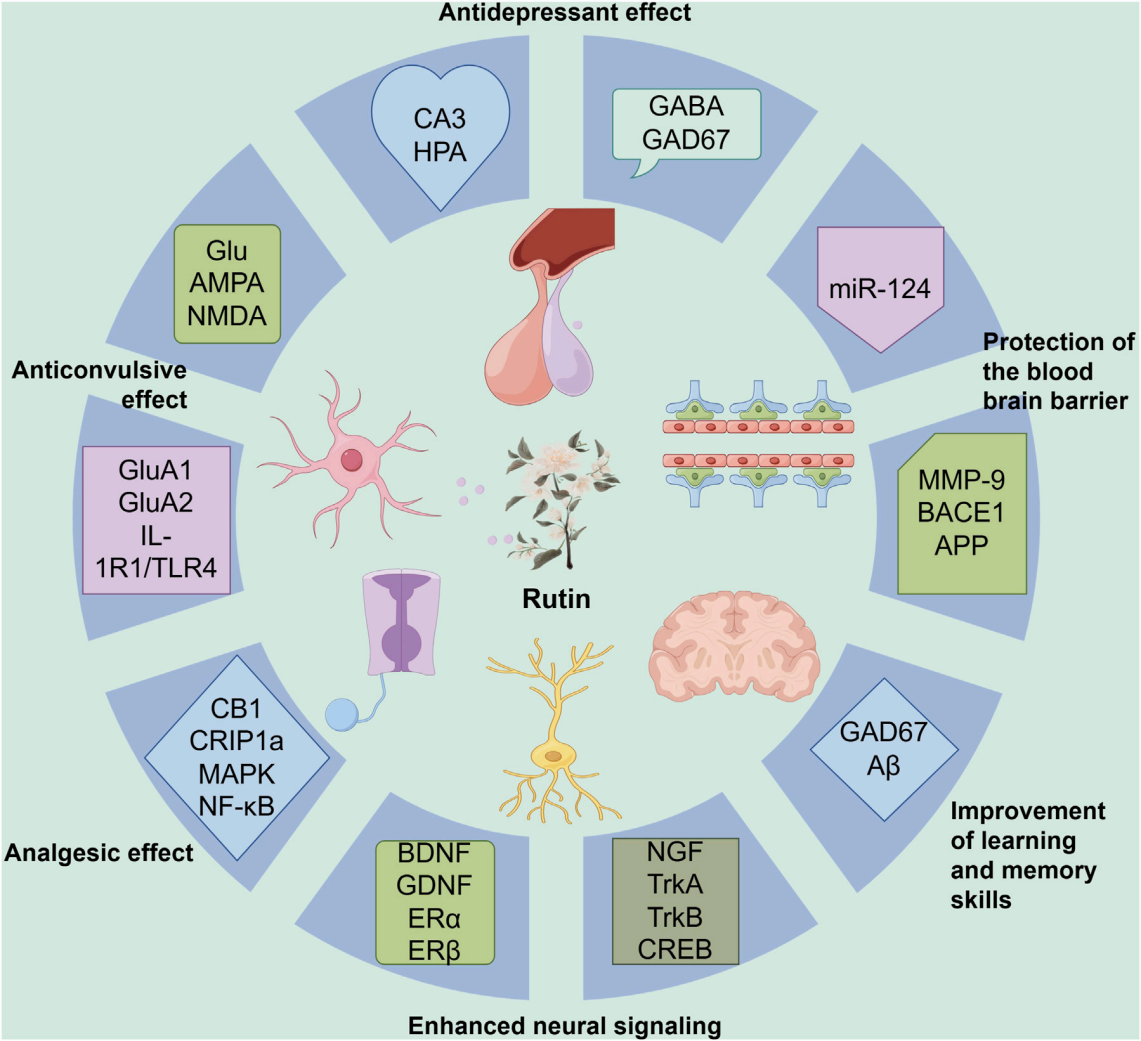
This figure illustrates the diverse neuroprotective effects of Rut and its underlying molecular mechanisms. Rut exhibits antidepressant effects by modulating the CA3 region and the HPA axis, as well as influencing GABA and GAD67. It demonstrates anticonvulsant properties through interactions with Glu, AMPA, NMDA receptors, and components like GluA1, GluA2, IL-1R1, and TLR4. Rut’s analgesic effects are linked to its interaction with CB1, CRIP1a, MAPK, and NF-κB pathways. The compound enhances neural signaling by regulating BDNF, GDNF, ERα, and ERβ. Additionally, rutin improves learning and memory skills via NGF, TrkA, TrkB, and CREB. The figure also highlights Rut’s role in protecting the BBB by modulating miR-124, MMP-9, BACE1, and APP. Furthermore, it shows Rut’s influence on GAD67 and Aβ in the context of neurological protection. Overall, this diagram provides a comprehensive overview of rutin’s multifaceted neuroprotective and neuromodulatory effects.

Gamma-Aminobutyric Acid (GABA) is an inhibitory neurotransmitter. The balance between GABA and excitatory neurotransmission is essential for the normal function of the brain. The study found that the content of GABA in plasma, cerebrospinal fluid and brain tissue samples of patients with depression decreased, the function of GABAR was defective, and the expression of GABA synthase GAD67 was downregulated. For GABA deficiency in depression, there are benzodiazepines that can enhance the function of GABAAR γ 2 subunit and tetrahydroperone preparations that act on GABAA receptor (Oriol et al. 2025). Wang X found Rut alleviated depressed mood, memory impairment and social impairment, ameliorated hippocampal neuronal damage and reduces GABA and acetylcholine (ACh) levels in depressed rats (Wang et al. 2025). In addition, Kessas K found that 200 mg/kg Rut intervened the aluminum poisoning model rats, and the GABA content in the brain tissue was significantly increased. Therefore, it is speculated that Rut can exert antidepressant effect by affecting GABAergic system (Kessas et al. 2024). The CNS and behavioral activity of Rut were tested by pore plate and thiopental sodium induced sleep time and motor activity in mice. Rut can inhibit the CNS by intraperitoneal administration. Studies have confirmed that Rut cannot have inhibitory activity of CNS due to the participation of GABA A receptor (Fernández et al. 2006). Foudah AI found that 14 days of treatment with Rut showed a modest antidepressant effect in reserpine-induced anxiety and depression in rats. Rut has shown antidepressant effects by reducing antioxidant activity and acetylcholinesterase (Foudah et al. 2022) (Figure 5; Table 4).

**TABLE 4:** Antidepressant effect of Rut.

Experimental models	Description	Observed effects	Key targets/Pathways	References
Dexamethasone-treated mice	Studies ventral tegmental area interneurons and the local synapses of GABA and glutamate projection neurons	Reveals characteristics of ventral tegmental area interneurons	GABA, glutamate neurons	Oriol et al. (2025)
Depression model rats	Explores the therapeutic potential of Rut in premenstrual depression	Rut shows potential therapeutic effects on premenstrual depression	Depression, neuroprotection	Wang et al. (2025)
Aluminum-induced neurotoxicity rat model	Examines the benefits of Rut on mitochondrial function and inflammation	Rut improves mitochondrial function and reduces inflammation	Mitochondrial function, inflammation	Kessas et al. (2024)
Flavonoid glycosides experimental model	Studies the central nervous system depressant effects of flavonoid glycosides	Flavonoid glycosides have a depressant effect on the central nervous system	Central nervous system depression	Fernández et al. (2006)
Reserpine-induced anxiety and depression in rats	Investigates the effects of Rut on anxiety and depression in rats	Rut improves anxiety and depression symptoms	Anxiety, depression	Foudah et al. (2022)

### 2.5 Anticonvulsant effect

Researchers conducted behavioral tests on psychomotor convulsion model mice, including motor coordination test, muscle strength test and long-term memory test, and found that Rut intervention mice had short-term anticonvulsant effect and Rut had no adverse reactions (Nieoczym et al. 2014). Nassiri-Asl M found that in the kainic acid-induced seizure rat model, Rut pretreatment can significantly reduce the severity of seizures and reverse hippocampal neuron loss (Nassiri-Asl et al. 2013). In addition, Researchers found that Rut can also regulate the level of glutamate, reduce the expression of glutaminase and NMDA receptor subunit glun2b, and increase the expression of excitatory amino acid transporter (EAAT), glutamine synthetase (GS) and AMPA receptor subunit GluA1 and GluA2 (Fikry et al. 2018).

Rut can also inhibit activated astrocytes, downregulate inflammatory molecules such as interleukin-1β (IL-1β), interleukin-6 (IL-6), tumor necrosis factor-α (TNF-α), high mobility group protein B1 (HMGB1), interleukin-1 receptor 1 (IL-1r1) and toll like receptor 4 (TLR-4), and upregulate the protein expression of anti-inflammatory molecule interleukin-10 (IL-10). In conclusion, the results suggest that Rut can reduce kainic acid-treated seizures and neuron loss by reducing glutamatergic hyperfunction and inhibiting IL-1r1/TLR4 related neuroinflammatory cascade (Chang et al. 2022). The effect of Rut injected into lateral ventricle on mild clonic seizures (MCS) and generalized tonic clonic seizures (GTC) induced by pentylenetetrazol (PTZ) was dose-dependent, and its mechanism was to increase the number of seizures. In addition, flumazenil pretreatment can eliminate the anticonvulsant effect of Rut during two seizures. These results indicate that Rut has anticonvulsant effect in the brain, which may be the positive allosteric regulation of GABA (a) receptor complex through the interaction at the benzodiazepine site (Nassiri-Asl et al. 2008). The convulsions of zebrafish were induced by benzotetrazole, and the behavior of zebrafish was studied, including latency, movement effect, color effect, fish cohesion, light/dark experiment. Antiepileptic drugs were used to interfere with the regional activities of fish, and Rut was used to treat them. It was found that the behavioral response of zebrafish returned to normal. These results indicate that Rut has a positive effect on the swimming behavior of zebrafish. Rut can increase the latency of zebrafish moving in the light room, suggesting that Rut plays its neuroprotective role by alleviating convulsive reaction (Dubey et al. 2015).

The anticonvulsant effect of Rut is closely related to its anti-inflammatory and cognitive improvement properties. Rut can inhibit activated astrocytes, downregulate the expression of inflammatory molecules (such as IL-1β, IL-6, TNF-α, etc.), and upregulate the expression of anti-inflammatory molecule IL-10, so as to improve cognitive ability. These mechanisms suggest that Rut exerts its neuroprotective effect against seizures by regulating the level of neurotransmitters, inhibiting neuroinflammation and improving cognitive ability (Figure 5; Table 5).

**TABLE 5:** Anticonvulsant effect of Rut.

Experimental models	Description	Observed effects	Key targets/Pathways	References
Acute seizure models in mice	Tests the effects of quercetin and Rut in some acute seizure models in mice	Rut shows short-term anticonvulsant effects with no adverse reactions	Anticonvulsant effects, neuroprotection	Nieoczym et al. (2014)
Kainic acid-induced seizure mice model	Studies the effects of Rut on oxidative stress in mice with kainic acid-induced seizures	Rut reduces oxidative stress and improves seizure outcomes	Oxidative stress, seizure improvement	Nassiri-Asl et al. (2013)
Paw inflammation model in mice	Examines the effects of Rut and meloxicam on paw inflammation in mice	Rut and meloxicam reduce paw inflammation by affecting sorbitol dehydrogenase activity	Paw inflammation reduction, sorbitol dehydrogenase activity	Fikry et al. (2018)
Kainic acid-treated rats model	Evaluates Rut’s effect on preventing seizures in kainic acid-treated rats	Rut prevents seizures by modulating glutamate levels, inflammation, and neuronal loss	Glutamate levels, inflammation, neuronal loss	Chang et al. (2022)
Intracerebroventricular administration in rats	Investigates the anticonvulsive effects of intracerebroventricular Rut administration	Rut demonstrates anticonvulsive effects when administered intracerebroventricularly	Anticonvulsive effects, GABA(A) receptor complex	Nassiri-Asl et al. (2008)
Zebrafish model	Explores the protective effect of Rut on cognitive function impairment caused by antiepileptic drugs on a zebrafish model	Rut protects cognitive functions from damage induced by antiepileptic drugs	Cognitive function protection	Dubey et al. (2015)

### 2.6 Analgesic effect

The generation of pain depends on pain signals uploaded to the CNS. Neurotransmitters and ion channels regulate the electrical activities of primary afferent nerves. Ionic glutamate receptors are coupled with ion channels and play an important role in mediating the transmission of pain signals in the CNS and PNS. Activation of glutamate receptors can induce the increase of NO and cGMP, and participate in the pain neuron response caused by peripheral inflammation (Alonso-Castro et al. 2017).

The antinociceptive effect of Rut in acetic acid-induced writhing test in mice was given 60 min before intraperitoneal injection of acetic acid. In the writhing test, the dose-response curve and the experimental effective dose 50 (ED50) of Rut in different dose combinations were measured. The results showed that Rut participated in two different mechanisms and had protective effect on writhing injury (Afsar et al. 2015). Rut has an effect on formalin induced paw inflammation in mice. Rut can significantly improve the licking time of mice on the first day, and has a good inhibitory effect on formalin induced paw inflammation pain in mice (Ferreira et al. 2021). In addition, studies have confirmed that Rut has peripheral and central antinociceptive activities. Zapata-Morales JR found that Rut synergistically produces analgesic effect by regulating opioid system, paracetamol and non steroidal anti-inflammatory drugs by down regulating p38 MAPK NF-κB and prostaglandins (Zapata-Morales JR et al. 2025). Su KY found that Rut significantly increased the expression of CB1 cannabinoid receptor interacting proteins A (crip1a) in the brain of mice (Su et al. 2014). Crip1a is an auxiliary protein of CB1 and can regulate the tension inhibition of voltage dependent calcium channels mediated by CB1 receptors. Tian R found that Rut had significant inhibitory effects on mechanical hyperalgesia, thermal hyperalgesia and cold hyperalgesia in diabetic rats, and the mechanism was related to inhibiting the activation of NF-κB and reducing the production of IL-6 and TNF-α in dorsal root ganglion (Tian et al. 2016).

The analgesic effect of Rut is induced by enhancing the antioxidant pool, reducing the levels of inflammatory factors (such as TNF-α) and IL-1β, inhibiting the expression of cyclooxygenase-2 (COX-2) and inducible nitric oxide synthase (iNOS), and regulating MAPK, NF-κB and Nrf-2/HO-1 signaling pathways (Figure 5; Table 6).

**TABLE 6:** Analgesic effect of Rut.

Experimental models	Description	Observed effects	Key targets/Pathways	References
Mouse model of visceral pain	Investigates the synergistic effects of naproxen and Rut in a mouse model of visceral pain	The combination of naproxen and Rut shows enhanced analgesic effects	Synergistic analgesic effects, visceral pain	Alonso-Castro et al. (2017)
Formalin test in rats	Evaluates the antipyretic, anti-inflammatory, and analgesic activity of Acacia hydaspica R. Parker and its phytochemical analysis	Rut contributes to the anti-inflammatory and analgesic activity observed	Anti-inflammatory, analgesic activity	Afsar et al. (2015)
Rat brain slices model	Examines Rut’s effects on glutamate uptake and excitotoxicity	Rut improves glutamate uptake and inhibits glutamate excitotoxicity	Glutamate uptake, glutamate excitotoxicity	Ferreira et al. (2021)
Formalin test in rats	Studies the synergistic interaction between Justicia spicigera extract and analgesics	Rut synergizes with analgesics to enhance pain relief	Synergistic interaction with analgesics	Zapata-Morales et al. (2025)
Forced swimming mouse model	Explores Rut’s effects on physical fatigue in a forced swimming mouse model	Rut attenuates physical fatigue	Physical fatigue reduction	Su et al. (2014)
Diabetic neuropathy rat model	Investigates Rut’s effects on diabetic neuropathy via the Nrf2 signaling pathway	Rut ameliorates diabetic neuropathy by lowering plasma glucose and decreasing oxidative stress	Diabetic neuropathy, Nrf2 signaling pathway	Tian et al. (2016)

### 2.7 Enhance neural signal transduction

Nerve regulation refers to the excitation, inhibition, or regulation of neurons or nerve signal transduction in the CNS, intestinal nervous system, and autonomic nervous system through various forms of stimulation enhancement. These may help to strengthen new nerve connections and enhance neural plasticity, and neurotrophic factors regulate nerve survival and proliferation in the nervous system (Zheng et al. 2025). Song K found that Rut pretreatment prevented the ethanol-induced decrease in protein level expression of nerve growth factor, glial cell line-derived neurotrophic factor (GDNF) and BDNF in HT22 cells (Song et al. 2015). Flavonoids improve the mechanism of DPN enhancing neurotrophic signal transduction. BDNF is associated with neurogenesis, neuronal maturation, survival and synaptic plasticity *in vivo*. BDNF combines with its receptor tropomyosin receptor kinase B (TrkB) to activate downstream signaling pathways, including phosphatidylinositol 3-kinase (PI3K)/AKT and mitogen activated protein kinase (MAPK), and plays its growth promoting role (Moghbelinejad et al. 2013). Liu H Found that Rut can enhance Erα ERβ, BDNF, The levels of nerve growth factor (NGF), tropomyosin receptor kinase A (TrkA), TrkB and cyclic adenosine monophosphate response element binding protein (p-CREB) in ovariectomized rats were measured and the cerebral ischemia/reperfusion injury in ovariectomized rats was improved. It has been improved that level of BDNF decreased in the hippocampus of depression patients and animal models (Liu et al. 2018). Koda T Found that Rut can improve memory damage induced by aβ25-35 by regulating BDNF signaling pathway in hippocampus (including Akt, ERK and CREB), and quantitative analysis of its crude extract found that Rut is a high content compound in Lespedeza, suggesting that Rut may participate in memory protection as the main component of Lespedeza in the process of up regulating BDNF signaling pathway in hippocampus (Koda et al. 2009) (Figure 5; Table 7).

**TABLE 7:** Rut can enhance neural signal transduction.

Experimental models	Description	Observed effects	Key targets/Pathways	References
Chronic pain model in rats	Examines the role of cGMP-dependent protein kinase I in the dorsal hippocampus in chronic pain-induced cognitive deficits	The kinase protects against synaptic plasticity and cognitive deficits induced by chronic pain	Synaptic plasticity, cognitive deficits	Zheng et al. (2025)
HT22 hippocampal neuronal cells model	Studies the effects of Rut on ethanol-induced oxidative stress	Rut upregulates neurotrophic factors and attenuates oxidative stress	Neurotrophic factors, oxidative stress	Song et al. (2015)
Beta-amyloid induced neurotoxicity in rats	Evaluates Rut’s activation of the MAPK pathway and BDNF gene expression	Rut activates the MAPK pathway and BDNF gene expression	MAPK pathway, BDNF gene expression	Moghbelinejad et al. (2014)
Cerebral ischemia-reperfusion injury model in ovariectomized rats	Investigates Rut’s effects on cerebral ischemia-reperfusion injury via estrogen-receptor-mediated signaling	Rut attenuates injury via BDNF-TrkB and NGF-TrkA signaling	BDNF-TrkB, NGF-TrkA signaling	Liu et al. (2018)
Toxicant-induced hippocampal injury model in rats	Studies Rut’s protective effects against toxicant-induced hippocampal injury	Rut suppresses microglial activation and pro-inflammatory cytokines	Microglial activation, pro-inflammatory cytokines	Koda et al. (2009)

### 2.8 Improve learning and memory ability

Ishola IO’s study found that Rut pretreatment can significantly reduce scopolamine induced nitrosation/oxidative stress and acetylcholinesterase activity in prefrontal cortex and hippocampus of mice. Rut can restore cognitive function of scopolamine induced amnesia by enhancing antioxidant defense system and cholinergic system (Ishola et al. 2020). Rut can reverse the learning and memory impairment of diabetic rats. 50 mg/kg Rut has obvious hypoglycemic effect on diabetic rats and non-diabetic rats, and long-term oral administration of Rut can induce cognitive enhancement in diabetic rats. Therefore, Rut is considered as a potential treatment for diabetic peripheral neuropathy (Hasanein et al. 2020). Studies have found that Rut can improve the energy metabolism of microglia in the brain of AD mice, increase the expression level of phagocytic receptors, and then restore and enhance the phagocytosis and clearance of a β by microglia, and improve ad related pathological and cognitive damage (Ouyang et al. 2022). Rut can also improve the spatial learning and memory ability of aging mice by inhibiting D-galactose-induced neuronal loss and apoptosis of hippocampal neurons (Guo et al. 2020). Studies have found that Rut can improve the cognitive function of SAMP8 mice fed with high-fat diet, and its mechanism is related to reducing the level of brain a β related protein and improving neuroinflammation (Swain et al. 2022). In the experiment of schizophrenic mice, Rut can prevent and treat ketamine induced spatial learning and memory impairment and improve their social ability by up regulating the expression of GAD67 in the striatum and prefrontal cortex (Oshodi et al. 2021).

Rut contains lecithin, triglyceride and other components that can directly promote the development of nervous system and brain cell activity, enhance children’s intellectual development and delay the cognitive decline of the elderly (De Araújo et al., 2023). At the same time, Rut can regulate capillary permeability, improve cerebral microcirculation and metabolic environment, and provide stable nutritional support for the brain (Thabet and Moustafa, 2018). In addition, Rut can enhance neuroprotective effect by promoting energy metabolism of microglia, which may have intervention effect on Alzheimer’s disease and other neurodegenerative diseases (Enogieru et al. 2018) (Figure 5; Table 8).

**TABLE 8:** Rut can improve learning and memory ability.

Experimental models	Description	Observed effects	Key targets/Pathways	References
Scopolamine-induced learning and memory impairments in mice	Examines Rut’s effects on scopolamine-induced learning and memory impairments	Rut enhances the antioxidant defense system and cholinergic signaling	Antioxidant defense, cholinergic signaling	Ishola et al. (2020)
Diabetes-induced deficits in rats	Investigates Rut’s effects on diabetes-induced deficits in learning, memory, and pain perception	Rut improves acquisition learning, retention memory, and pain perception	Learning, memory, pain perception	Hasanein et al. (2020)
Alzheimer’s disease model in rats	Studies the effects of co-delivering miR-124 and Rut using DNA nanoflowers	Rut and miR-124 synergistically improve Alzheimer’s disease outcomes	miR-124, BDNF signaling	Ouyang et al. (2022)
D-galactose-induced oxidative stress in mice	Evaluates the preventive effects of Apocynum venetum polyphenols on oxidative stress	Rut reduces neuronal loss and apoptosis	Oxidative stress, neuronal protection	Guo et al. (2020)
High-fat diet fed-streptozotocin-induced diabetic encephalopathy in rats	Evaluates the preventive effects of Apocynum venetum polyphenols on oxidative stress	Rut improves cognitive function by regulating oxidative stress and neuroinflammation	Cognitive function, neuroinflammation	Swain et al. (2022)
Ketamine-induced schizophrenia-like behavior in mice	Investigates Rut’s effects on ketamine-induced behavior changes	Rut prevents and reverses schizophrenia-like behavior	Glutamic acid decarboxylase, cholinergic pathways	Oshodi et al. (2021)
Aminochrome neurotoxicity model	Studies Rut’s protective effects against aminochrome neurotoxicity	Rut provides protection against neurotoxicity	Neuroprotection	De Araújo et al. (2023)
Acrylamide- or gamma radiation-induced brain injury in rats	Examines Rut’s protective effects against brain injury	Rut acts through the PI3K/AKT/GSK-3β/NRF-2 signaling pathway	PI3K/AKT/GSK-3β/NRF-2 pathway	Thabet and Moustafa (2018)
Neurodegenerative disorders model	Explores Rut’s antioxidant effects and implications for neurodegenerative disorders	Rut shows potent antioxidant effects relevant to neurodegenerative disorders	Antioxidant effects	Enogieru et al. (2018)

**TABLE 9:** Rut can protect blood brain barrier.

Experimental models	Description	Observed effects	Key targets/Pathways	References
Delayed thrombolysis model in animals	Examines acupuncture’s regulation of astrocyte neurotoxic polarization	Protects blood-brain barrier integrity through the ERK1/2/Cx43 axis	ERK1/2/Cx43 axis, blood-brain barrier	Zhang et al. (2025)
Photothrombotic focal ischemic model in rats	Studies Rut’s effects on functional outcome by reducing matrix metalloproteinase-9 levels	Rut improves functional outcome via reducing MMP-9	MMP-9	Jang et al. (2014)
Mouse models of Alzheimer’s disease	Investigates the cognitive improvement effects of PF-04957325 via modulating neuroinflammation	PF-04957325 improves cognition by modulating neuroinflammation	Neuroinflammation	Guo TY et al. (2025)
Brain-targeted nanoliposome loaded with Rut	Examines the preparation and pharmacokinetics of brain-targeted Rut nanoliposomes	Enhanced delivery and efficacy of Rut in the brain	Brain-targeted delivery	Wu et al. (2024)

### 2.9 Protect blood brain barrier

BBB is a kind of barrier composed of vascular endothelial cells, basement membrane and astrocytes, which is located in the cerebrovascular system. Its main function is to limit the damage of external substances to the brain, only allow nutrients, oxygen and other essential substances to pass through, filter out harmful substances and protect the brain from harmful substances (Zhang et al. 2025).

Rut can reduce the damage of BBB caused by cerebral thrombosis and local ischemia, and improve acute ischemic stroke. The mechanism is partly through inhibiting the expression and activity of matrix metalloproteinase-9 (MMP-9) (Jang et al., 2014). Rut can cooperate with miR-124 to inhibit the expression of BACE1 and app, thus strongly inhibiting the production of Aβ. The nano system can promote the long-term circulation of miR-124 *in vivo*, promote its BBB permeability and neuron targeting, and significantly increase the content of miR-124 in hippocampus of APP/PS1 mice, which has good therapeutic effect *in vivo*. This biologically derived therapeutic system shows the prospect of being used as a biocompatible nanodrug in the treatment of ad (Guo TY et al., 2025). Rut liposomes were prepared by film dispersion method, and the preparation conditions were optimized by response surface methodology. Then transferrin (TF) was introduced into liposomes through covalent modification to prepare TF Rut liposomes, indicating that TF Rut lip has brain targeting, which may improve the efficacy of Rut in the treatment of brain diseases (Wu et al. 2024) (Figure 5; Table 9).

## 3 Discussion and perspectives

Rut is emerging as a highly promising neuroprotective agent, demonstrating a wide array of mechanisms that equip it to address multiple facets of neurological disorders. Its antioxidant properties are particularly vital in the context of neurological diseases, many of which are underpinned by oxidative stress. The hydroxyl groups on the flavonoid core of Rut are key to its antioxidant properties. The conjugated system formed by the benzene rings and oxygen atoms in the structure enables Rut to stabilize free radicals and scavenge reactive oxygen species such as superoxide anions, hydroxyl radicals, and peroxyl radicals, thereby reducing oxidative damage to the body. The glycosidic moiety influences its antioxidant activity by enhancing its solubility in water and facilitating its transport and absorption within biological systems, allowing it to exert antioxidant effects in both hydrophilic and hydrophobic environments (Agarwal et al. 2025; L Suraweera et al. 2020). In the fields of neuroinflammation, Rut’s role is equally significant. It can inhibit the activity of inflammatory mediators such as cyclooxygenase and lipoxygenase. Its structural components interact with key enzymes and signaling molecules involved in the inflammatory response, blocking inflammatory signaling pathways and reducing the production and release of inflammatory cytokines like interleukins and tumor necrosis factor-alpha. Additionally, its antioxidant properties help mitigate oxidative stress-induced inflammation (Habtemariam, 2016; Budzynska et al. 2019). Moreover, Rut also can regulate the delicate balance of apoptosis-related genes, it can interact with a variety of signaling pathways and molecular targets during development. It also can regulate cell cycle related proteins, induce cell cycle arrest and prevent cell proliferation. It promotes cell apoptosis by activating apoptosis signaling pathway. This is crucial for preserving neuronal integrity and function (Wen and Hu, 2024; Zhang et al. 2021).

Beyond these mechanisms, Rut has shown antidepressant, anticonvulsant, and analgesic effects. These multifaceted effects can be attributed to its ability to regulate the HPA axis, which is often dysregulated in stress-related disorders, as well as its modulation of neurotransmitter systems and inflammatory responses (Chen et al. 2022). Rut can alleviate symptoms associated with depression and anxiety while also providing relief in seizure disorders and pain conditions by enhancing GABAergic transmission or modulating the release of pro-inflammatory cytokines (Emudainohwo et al., 2023). Rut’s impact on neural signal transduction is another key aspect of its neuroprotective profile. It can upregulate neurotrophic factor BDNF, which plays a crucial role in supporting the survival and growth of neurons, enhancing synaptic plasticity, and promoting neurogenesis (Çelik et al. 2020). The activation of downstream signaling pathways like PI3K/AKT and MAPK by BDNF further amplifies, Rut’s beneficial effects on learning and memory (Ola et al. 2015). Moreover, Rut has been found to protect the BBB by inhibiting the expression and activity of MMP-9 (Sun et al. 2025).

Despite these extensive and promising neuroprotective effects, Rut’s journey to clinical application faces some challenges, such as its relatively low bioavailability, chemically unstable under certain conditions, including light, heat, and pH, pharmacokinetic limitations, interact with other medication could either reduce Rut’s effectiveness or increase the risk of adverse effects and its safety profile with long-term use or at high doses (Ozkan et al. 2020; Goyal and Verma et al., 2023). These limitations have dampened their therapeutic potential in humans despite its efficacy in preclinical models. However, the development of advanced drug delivery systems, nano systems or liposomes with brain targeting properties, Genetic engineering and metabolic engineering techniques and combine with other bioactive substances could markedly enhance Rut’s bioavailability. These innovative formulations could facilitate the efficient delivery of Rut across the BBB to target tissues, thereby maximizing its therapeutic effects (Malekpour et al. 2025).

In conclusion, Rut boasts a multitude of neuroprotective mechanisms that position it as a versatile and potent therapeutic agent for neurological disorders. While further research is needed to optimize its delivery and overcome its bioavailability limitations, the existing evidence underscores Rut’s immense potential. With continued scientific efforts, Rut could evolve into a cornerstone of neuroprotective therapy, offering new hope for patients suffering from a wide spectrum of neurological diseases.

## Glossary

AD: Alzheimer’s disease
ARE: Antioxidant response element
Arg1: Arginase 1
Aβ: Amyloid-beta
Bax: B-cell lymphoma-2 associated X protein
BBB: blood-brain barrier
Bcl-2: anti apoptotic gene B-cell lymphoma-2
BDNF: brain-derived neurotrophic factor
CAT: catalase
CNS: Central nervous system
COX-2: cyclooxygenase-2
CREB: cAMP response element binding protein
DG: dentate gyrus
ERK: extracellular signal regulated kinase
GABA: Gamma-Aminobutyric Acid
GDNF: glial cell line derived neurotrophic factor
GSH-PX: glutathione peroxidase
HO-1: Heme Oxygenase-1
IL-10: interleukin-10
IL-1β: interleukin-1β
IL-6: interleukin-6
iNOS: inducible nitric oxide synthase
JNK: c-Jun N-terminal kinase
LPS: Lipopolysaccharide
MAPK: mitogen activated protein kinas
MDA: malondialdehyde
MMP-9: matrix metalloproteinase-9
MPP(+): 1-methyl-4-phenylpyridinium ion
NF-κB: nuclear factor kappa B
NGF: nerve growth factor
Nrf2: nuclear factor red cell 2 related factor 2
PCC: pheochromocytoma cells
P-CREB: cyclic adenosine monophosphate response element binding protein
PHB2: Prohibitin 2
PI3K: phosphatidylinositol 3-kinase
RhoA/ROCK: Ras homolog family member A/Rho-associated coiled-coil kinase
ROS: reactive oxygen species
Rut: Rutin
SOD: superoxide dismutase
TLR4: Toll-like receptor 4
TNF-α: tumor necrosis factor-α
TrkA: tropomyosin receptor kinase A
TrkB: tropomyosin receptor kinase B
